# Supplementary material for: Bibliometric Analysis of the Knowledge Base and Future Trends on Sarcopenia from 1999–2021
Source: Int J Environ Res Public Health. 2022 Jul 21;19(14):8866. doi: 10.3390/ijerph19148866 (PMC9320125; doi:10.3390/ijerph19148866)
Supplement: Supplementary file 1 [file ijerph-19-08866-s001.zip › ijerph-1698584-supplementary.pdf]

Table S1. The top 10 most productive countries in the sarcopenia research field.

Countries	Documents	Number of papers per trillion GDP	Citations	TLS
USA	690	33.22	47260	551
China	534	36.10	13687	172
Japan	524	104.99	16829	104
Italy	358	191.14	29605	478
South Korea	315	194.15	9220	100
UK	250	99.28	22401	442
Spain	185	144.36	17890	335
Germany	179	47.06	18940	297
Brazil	173	120.44	3587	100
France	171	65.39	22583	330

GDP is the gross domestic product; TLS is the total link strength. Data on GDP and percentage of aged 65 or above of countries were obtained from the World Bank (http://data.worldbank.org/indicator).

Table S2. Correlation between the number of publications in sarcopenia and GDP or percentage of old adults of the countries.

	GDP	Percentage of the population aged 65 or above
**Number of publications**	$r = 0.851$	$r = 0.338$
	$P < 0.001$	$P = 0.003$

GDP is the gross domestic product, P value less than 0.05 was considered statistically significant.

Table S3. The top 10 most prolific organizations in the sarcopenia research field.

Organizations	Country	NP	NC	TLS
Catholic University of the Sacred Heart	Italy	92	16032	119
University of Melbourne	Australia	73	1633	66
Sichuan University	China	65	1155	1
University of Liege	Belgium	55	5208	62
University of Southampton	UK	51	5609	109
Yonsei University	South Korea	51	1032	7
University of Florida	USA	49	4794	101
National Center for Geriatrics and Gerontology	Japan	49	3836	58
Seoul National University	South Korea	48	2187	31
Maastricht University	Netherlands	41	2330	32

NP is the number of publications, NC is the number of citations, TLS is total link strength.

Table S4. The top 10 most productive authors in the sarcopenia research field.

Authors	Affiliations	NP	NC	TLS
Landi F	Catholic University of the Sacred Heart	70	14706	322
Marzetti E	Catholic University of the Sacred Heart	49	3017	221
Cesari M	University of Milan	48	3297	175
Bruyere O	University of Liege	44	4805	147
Beaudart C	University of Liege	42	1995	132
Bernabei R	Catholic University of the Sacred Heart	40	3180	187
Cooper C	University of Southampton	39	5225	119
Sayer AA	Newcastle University	39	4881	62
Calvani R	Catholic University of the Sacred Heart	39	2036	196
Reginster JY	University of Liege	32	2091	123

NP is the number of publications, NC is the number of citations, TLS is total link strength.

Table S5. The top 25 most frequently used keywords on sarcopenia research.

Keywords	Occurrence	TLS
sarcopenia	2573	15291
muscle mass	1053	7229
skeletal-muscle	937	5355
older-adults	838	5867
body-composition	835	5706
prevalence	729	5165
age	640	3949
mortality	516	3599
obesity	487	3394
strength	486	3421
frailty	420	2773
consensus	399	2641
mass	393	2689
health	349	2559
muscle strength	328	2335
association	322	2332
risk	309	2161
exercise	291	1950
survival	287	1818
muscle	276	1724
men	274	2044
women	265	2025
adults	262	1829
physical-activity	253	1716
physical performance	241	1767

TLS is total link strength.

Table S6. The most highly cited references in the sarcopenia research field.

Rank	Title	First author	Journals	PY	Citations
1	Sarcopenia: European consensus on definition and diagnosis	Cruz-Jentoft AJ	*Age and Aging*	2010	6271
2	Sarcopenia: revised European consensus on definition and diagnosis	Cruz-Jentoft AJ	*Age and Aging*	2019	2615
3	Sarcopenia in Asia: Consensus Report of the Asian Working Group for Sarcopenia	Chen LK	*Journal of the American Medical Directors Association*	2014	1949
4	Low relative skeletal muscle mass (sarcopenia) in older persons is associated with functional impairment and physical disability	Janssen I	*Journal of the American Geriatrics Society*	2002	1875
5	Sarcopenia: An Undiagnosed Condition in Older Adults. Current Consensus Definition: Prevalence, Etiology, and Consequences. International Working Group on Sarcopenia	Fielding RA	*Journal of the American Medical Directors Association*	2011	1675
6	Age-associated changes in skeletal muscles and their effect on mobility: an operational diagnosis of sarcopenia	Lauretani F	*Journal of Applied Physiology*	2003	1121
7	Aging and sarcopenia	Doherty TJ	*Journal of Applied Physiology*	2003	1072
8	The FNIH Sarcopenia Project: Rationale, Study Description, Conference Recommendations, and Final Estimates	Studenski SA	*Journals of Gerontology, Series A: Biological Sciences and Medical Sciences*	2014	1017
9	Consensus definition of sarcopenia, cachexia and pre-cachexia: Joint document elaborated by Special Interest Groups (SIG) "cachexia-anorexia in chronic wasting diseases" and "nutrition in geriatrics"	Muscaritoli M	*Clinical Nutrition*	2010	961
10	Prevalence of and interventions for sarcopenia in ageing adults: a systematic review. Report of the International Sarcopenia Initiative (EWGSOP and IWGS)	Cruz-Jentoft AJ	*Age and Aging*	2014	905

PY is the publication year.
